# A long non-coding RNA signature for predicting survival in patients with colorectal cancer

**DOI:** 10.18632/oncotarget.23431

**Published:** 2017-12-19

**Authors:** Yi-Lin Wang, Jun Shao, Xiaohong Wu, Tong Li, Ming Xu, Debing Shi

**Affiliations:** ^1^ Department of Hepatic Surgery, Fudan University Shanghai Cancer Center, Department of Oncology, Shanghai Medical College, Fudan University, Shanghai 200032, China; ^2^ Department of Colorectal Surgery, Fudan University Shanghai Cancer Center, Department of Oncology, Shanghai Medical College, Fudan University, Xuhui District, Shanghai 200032, China; ^3^ Department of General Surgery, Tongren Hospital, Shanghai Jiao Tong University School of Medicine, Changning District, Shanghai 200336, China; ^4^ Department of General Surgery, The Affiliated Yixing Hospital of Jiangsu University, Yixing, Jiangsu 214200, China; ^5^ Department of General Surgery, Jinan Fourth People's Hospital, Jinan 250031, China

**Keywords:** long non-coding RNA, prognosis, colorectal cancer

## Abstract

Dysregulation of long non-coding RNA (lncRNA) plays important roles in cancer development and progression. In this work, we attempted to develop a lncRNA signature to improve prognosis prediction of colorectal cancer. A comprehensive analysis for the lncRNA expression and corresponding clinical information of 344 colorectal patients has been performed based on the data from The Cancer Genome Atlas (TCGA). We randomly divided TCGA data into a training set (*n* = 172) and a testing set (*n* = 172). A four-lncRNA signature has been established which was significantly associated with the overall survival of colorectal cancer patients. Based on the four-lncRNA signature, the training set can be classified into high-risk and low-risk groups with significantly different survival. The result can be further validated in the testing dataset and another independent dataset. Further analyses suggested that the prognostic power of the four-lncRNA signature was independent of other clinical variables. The identification of lncRNA signature indicated that lncRNAs could be novel independent biomarkers for predicting the survival in patients with colorectal cancer.

## INTRODUCTION

Colorectal cancer (CRC) is the third most common malignancy, and is the major cause of cancer-related death worldwide [1, 2]. The incidence of colorectal cancer is gradually increasing in the developed areas. To date, surgery followed by adjuvant therapy is still the most common option for CRC patients. Despite an improved understanding of the molecular mechanism of CRC, the overall survival (OS) of CRC patients has not been dramatically improved and the four-year survival rate remains very low [3]. It is an urgent need to identify novel independent biomarkers for the diagnostic and prognosis of CRC.

With the advancements of transcriptome profiling, the roles of long non-coding RNAs (lncRNAs) have received great attention in the development of human cancer researches. LncRNAs are an important category of non-coding RNAs with little or no protein-coding capacity [4, 5]. It has been documented that lncRNAs play important roles in regulating gene expression at transcriptional, posttranscriptional and epigenetic levels [4, 6–8]. Moreover, lncRNAs can participate in various biological processes and pathways, such as cell growth and immune response [7, 9, 10]. Recently, many lncRNAs have been examined to play critical oncogenic or tumor suppressive roles in various types of cancers [11–14]. Furthermore, several lncRNAs have been identified to be novel independent biomarkers for cancer prognosis [15–20]. As for colorectal cancer, recent studies have also revealed that some lncRNAs, such as *PANDR*, *AFAP1-AS1* and *TUG1*, are dysregulated in CRC patients and play important role in the tumorigenesis [21–25].

We here attempted to develop a lncRNA signature to improve prognosis prediction of CRC. We identified a four-lncRNA signature by using the sample-splitting method. Our results demonstrated the four-lncRNA signature can provide a novel insight into the understanding of the underlying molecular mechanism of CRC.

## RESULTS

### Identification of prognostic lncRNAs from the training dataset

The 344 CRC patients were randomly divided into a training dataset (*n* = 172) and a testing dataset (*n* = 172). At first, we identified the prognostic lncRNAs from the training set. A univariate Cox regression analysis was performed to evaluate the association between lncRNA expression and overall survival of CRC patients. Based on the threshold of *P-value* < 0.01, four lncRNAs were identified to be significantly correlated with overall survival of CRC patients. The detailed information of these four lncRNAs was showed in Table 1. Positive coefficients represent that higher expression profiles were associated with shorter overall survival (*SPRY4-IT1*), whereas negative coefficients represent that higher expression level of lncRNA expression was associated with longer survival (*LINC01133*, *Loc554202* and *RP11-727F15.13*).

**Table 1:** The detailed information of four prognostic lncRNAs significantly associated with overall survival in patients with CRC

Gene symbol	*P* value^a^	Hazard ratio^a^	Coefficient^b^
*SPRY4-IT1*	3.71E–04	1.637	0.322
*RP11-727F15.13*	2.88E–03	0.746	–0.231
*Loc554202*	6.38E–03	0.560	–0.134
*LINC01133*	1.39E–04	0.751	–0.336

^a, b^Derived from the univariate and multivariate Cox regression analyses in CRC patients of the training dataset.

### A four-lncRNA signature for predicting overall survival of CRC patients

These four lncRNAs were analyzed using a multivariate Cox regression analysis to establish a lncRNA signature for predicting patients’ overall survival. We constructed a risk-score formula by integrating the lncRNA expressions and corresponding estimated regression coefficient derived from above multivariate Cox regression analysis, as follows: Risk score = (0.322 × expression value of *SPRY4-IT1*) + (–0.134 × expression value of *Loc554202*) + (–0.336 × expression value of *LINC01133*) + (–0.231 × expression value of *RP11-727F15.13*). We calculated four-lncRNA signature risk score for each CRC patient, and ranked them according to risk score values. These 172 CRC patients can be divided into a high-risk group (*n* = 90) and a low-risk group (*n* = 82) using the median risk score as the threshold.

**Figure 1:**
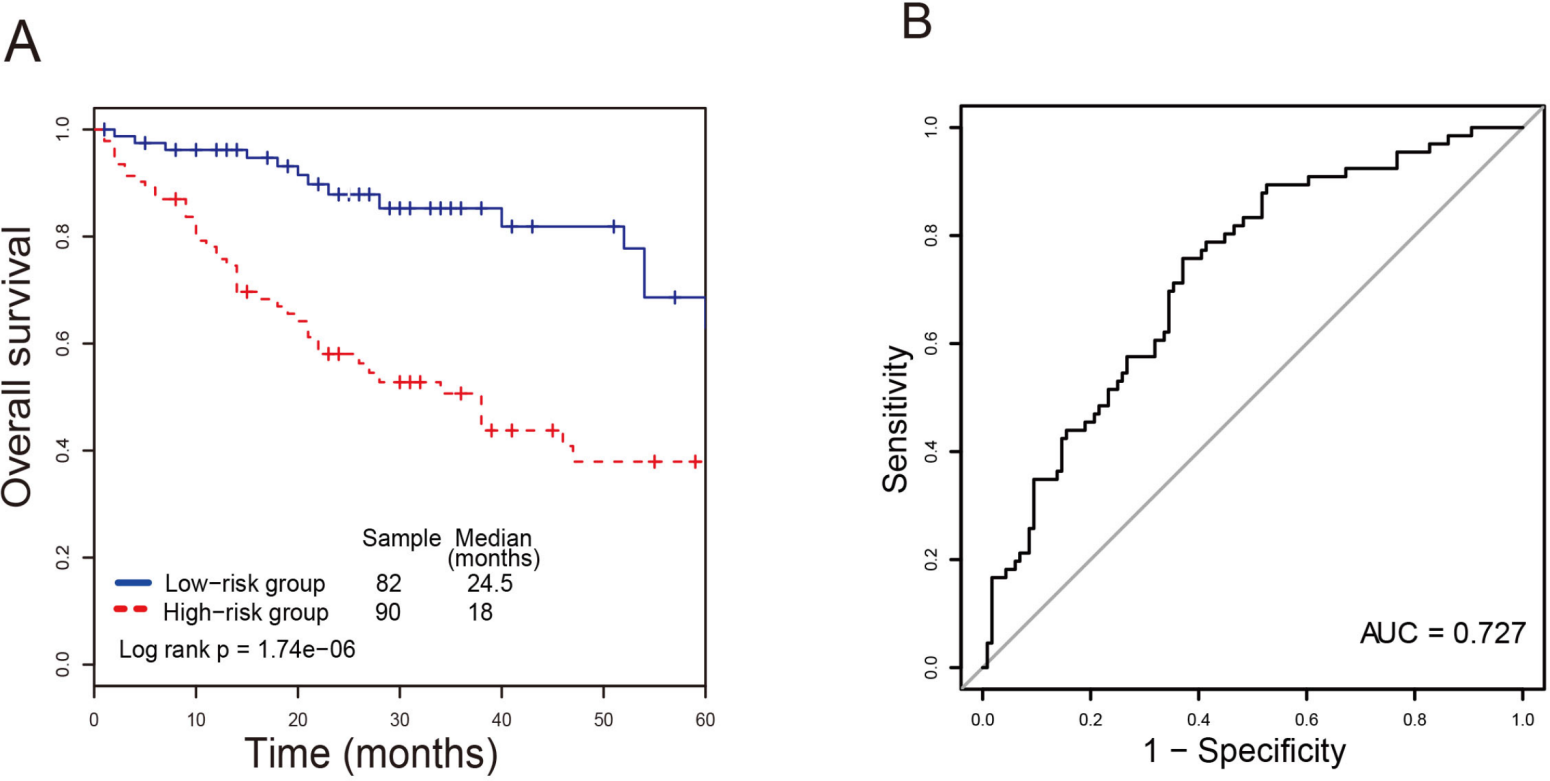
The four-lncRNA signature in prognosis of survival of CRC patients in the training dataset (**A**) The Kaplan-Meier curves of overall survival between high-risk and low-risk patients in the training dataset. (**B**) The ROC curve for survival prediction by the four-lncRNA signature within four years as the defining point.

A significant difference of overall survival between the high-risk group and low-risk group was observed (*P-value* = 1.74E-06; Figure 1A). It is obvious that CRC patients in the high-risk group had significantly shorter survival (median 18 months) than those in the low-risk group (median 24.5 months). The time-dependent ROC curve analysis achieved an AUC of 0.727 at the overall survival of five years (Figure 1B), suggesting a competitive performance of the four-lncRNA signature for survival prediction. The lncRNA risk score were significantly associated with overall survival of CRC patients using the univariate Cox regression analysis (Table 2).

**Table 2:** Univariate and multivariate cox regression analyses in each dataset

Variables	Univariate analysis			Multivariate analysis		
	HR	95% CI of HR	*P* value	HR	95% CI of HR	*P* value
**Training dataset (*n* = 172)**						
Four-lncRNA risk score						
Low risk/High risk	17.25	1.28–29.40	1.37E-05	21. 62	1.34–32.28	6.83E-05
Age						
≤65/>65	1.74	0.54–4.91	0.006	1.53	0.37–3.70	0.0042
Gender						
Female/Male	0.826	0.38–4.61	0.62	0.91	0.51–2.78	0.73
Stage						
II	1 (reference)			1 (reference)		
III/IV	2.18	0.58–4.71	0.012	2.24	0.36–4.95	0.01
**Testing dataset (*n* = 172)**						
Four-lncRNA risk score						
Low risk/High risk	3.33	1.42–5.71	3.01E-03	2. 59	1.07–5.89	6.48E-03
Age						
≤65/>65	1.20	0.48–5.74	0.006	183	0.45–3.24	0.008
Gender						
Female/Male	1.49	0.43–2.95	0.67	1.23	0.44–3.16	0.62
Stage						
II	1 (reference)			1 (reference)		
III/IV	1.65	0.55–5.44	0.021	1.49	0.42–5.11	0.033
**Entire dataset (*n* = 344)**						
Four-lncRNA risk score						
Low risk/High risk	5.56	2.81–11.72	6. 8E-04	4.98	2.54–9.88	8.42E-04
Age						
≤65/>65	1.12	0.603–4.03	0.01	1.29	0.63–5.48	0.01
Gender						
Female/Male	0.74	0.51–1.95	0.35	1. 45	0.56–2.9	0.54
Stage						
II	1 (reference)			1 (reference)		
III/IV	2.92	1.26–5.85	0.01	1.66	0.74–5.42	0.01

### Validation of the four-lncRNA signature for survival prediction in the testing dataset and another independent dataset

**Figure 2:**
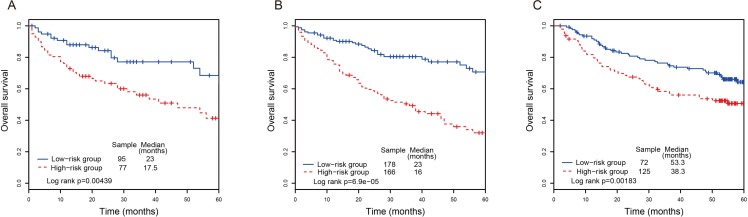
The Kaplan-Meier curves of overall survival between high-risk and low-risk patients in the testing, entire dataset and another independent dataset (**A**) The Kaplan-Meier curves for the testing dataset. (**B**) The Kaplan-Meier curves for the entire dataset. (**C**) The Kaplan-Meier curves for the dataset from Gene Expression Omnibus database.

We confirmed our results using the testing set. Using the same risk score formula, 172 CRC patients can be classified into a high-risk group (*n* = 77) and a low-risk group (*n* = 95) with the same cutoff point derived from the training dataset. The result showed that a significant difference of overall survival between the high-risk group and the low-risk group (*P-value* = 0.00439, median 17.5 months *vs*. 23 months; Figure 2A). The AUC value in the testing set was 0.712 at the overall survival of four years, and the lncRNA risk score was significantly associated with patients’ overall survival (Table 2). Next, we performed the same analysis in the entire TCGA CRC dataset. similar results were obtained. The lncRNA signature can classify 344 CRC patients into a high-risk group (*n* = 166) and a low-risk group (*n* = 178) with significant difference of overall survival (*P-value* = 6.9E-05, median 16 months *vs*. 23 months; Figure 2B). The AUC value in the entire set was 0.721 at the overall survival of four years. Further analysis indicated that lncRNA risk score was significantly associated with CRC patients’ overall survival in the entire TCGA CRC dataset (Table 2). We further validated our lncRNA signature in an independent CRC data (GSE14333). As shown in Figure 2C, lncRNA signature can effectively predict overall survival in CRC patients. A significant difference of overall survival between the high-risk group (*n* = 125) and the low-risk group (*n* = 72) was observed (*P-value* = 0.0183, median 38.3 months *vs*. 58.3 months).

### Independence of the lncRNA signature for survival prediction from other clinical variables

**Figure 3:**
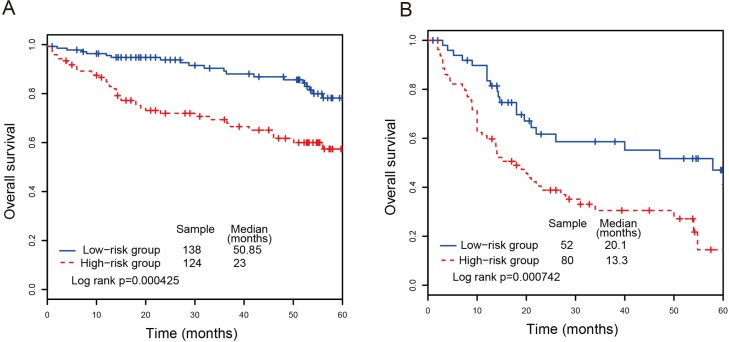
Survival analyses of all CRC patients stratified by age and tumor stage with the four-lncRNA signature (**A**) The Kaplan-Meier curves for the younger dataset. (**B**) The Kaplan-Meier curves for the elder dataset.

**Figure 4:**
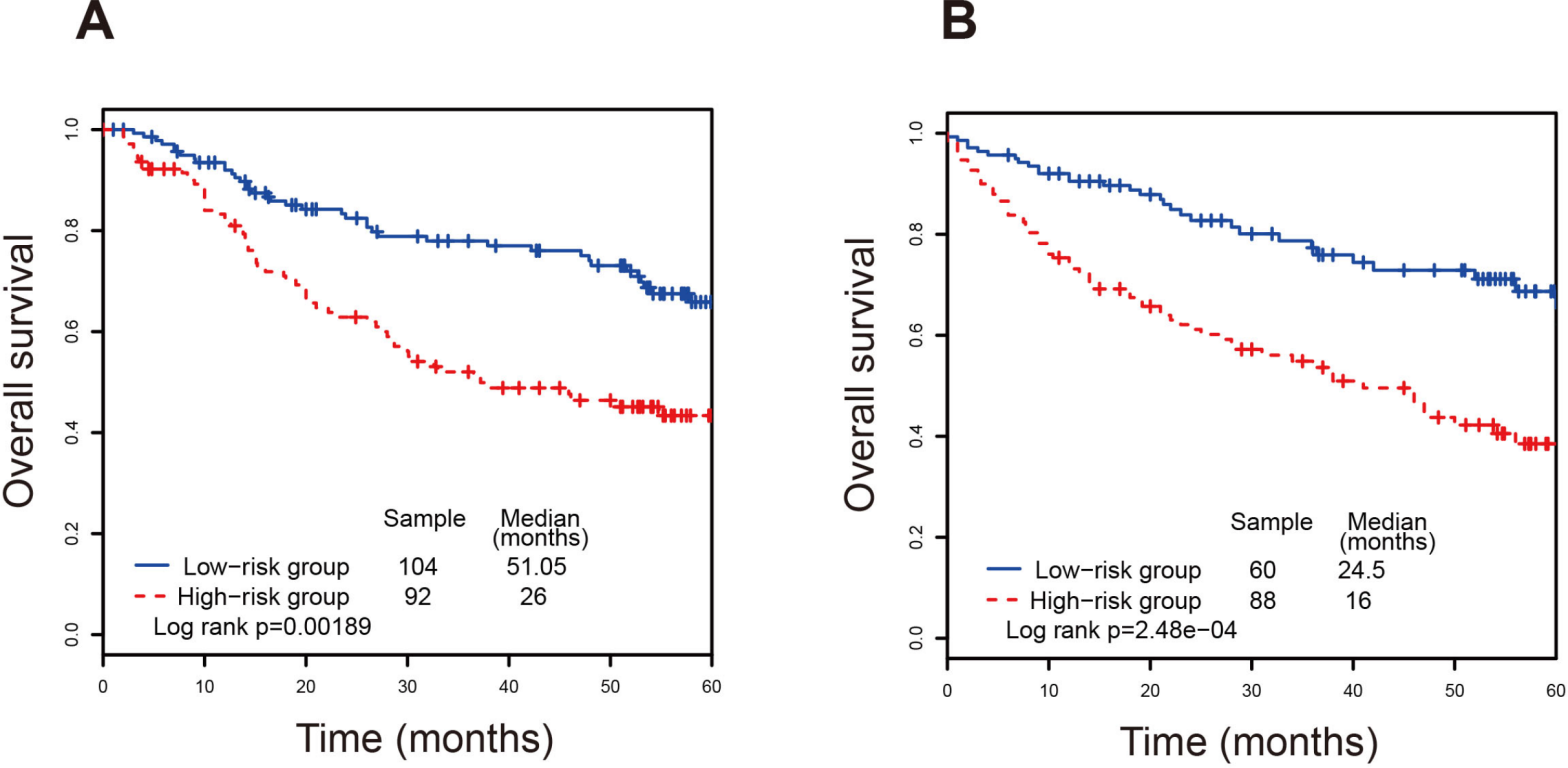
Survival analyses of all CRC patients stratified by tumor stage with the four-lncRNA signature (**A**) The Kaplan-Meier curves for the early stage dataset. (**B**) The Kaplan-Meier curves for the late stage dataset.

We examined whether the prognostic power of the lncRNA signature was independent of other clinical variables, such as age, gender, subtype and tumor stage. The multivariate Cox regression analyses were performed, and the results suggested that the lncRNA risk score was also significantly associated with overall survival. The lncRNA signature still maintained a significant association with overall survival after adjustment for other clinical variables (Table 2). The result showed that patient age and tumor stage were significantly associated with overall survival. A series stratified analyses have been performed according to age and tumor stage, respectively. At first, all CRC patients were stratified into a younger group (*n* = 132, age < 65) and an elder group (*n* = 212, age ≥ 65). The lncRNA signature can divided the younger group into a high-risk subgroup (*n* = 85) and a low-risk subgroup (*n* = 47) with significant difference of survival (*P-value* = 0.00416, median 23 months *vs*. 50.85 months; Figure 3A). As for the elder group, the four-lncRNA signature was also able to classify them into a high-risk subgroup (*n* = 147) and a low-risk subgroup (*n* = 65) with significantly different survival (*P-value* = 0.00742, median 13.3 months *vs*. 20.1 months; Figure 3B). Next, all CRC patients were stratified by tumor stage into an early subgroup (stage I and II, *n* = 196) and a late subgroup (stage III and IV, *n* = 148), respectively. The result of stratified analysis showed effective prognostic power in both early subgroup and late subgroup. As shown in Figure 4A, patients in the early subgroup can be divided into a high-risk group (*n* = 92) with shorter survival and a low-risk group (*n* = 104) with longer survival (*P-value* = 0.00189, median 26 months *vs*. 51.05 months). Similar results were obtained in the late subgroup (*P-value* = 2.48E-04, median 16 months *vs*. 24.5 months; Figure 4B). These result demonstrated that the prognostic ability of lncRNA signature is independent of other clinical variables for the prediction of survival in CRC patients.

### Functional implications of the prognostic lncRNAs

**Figure 5:**
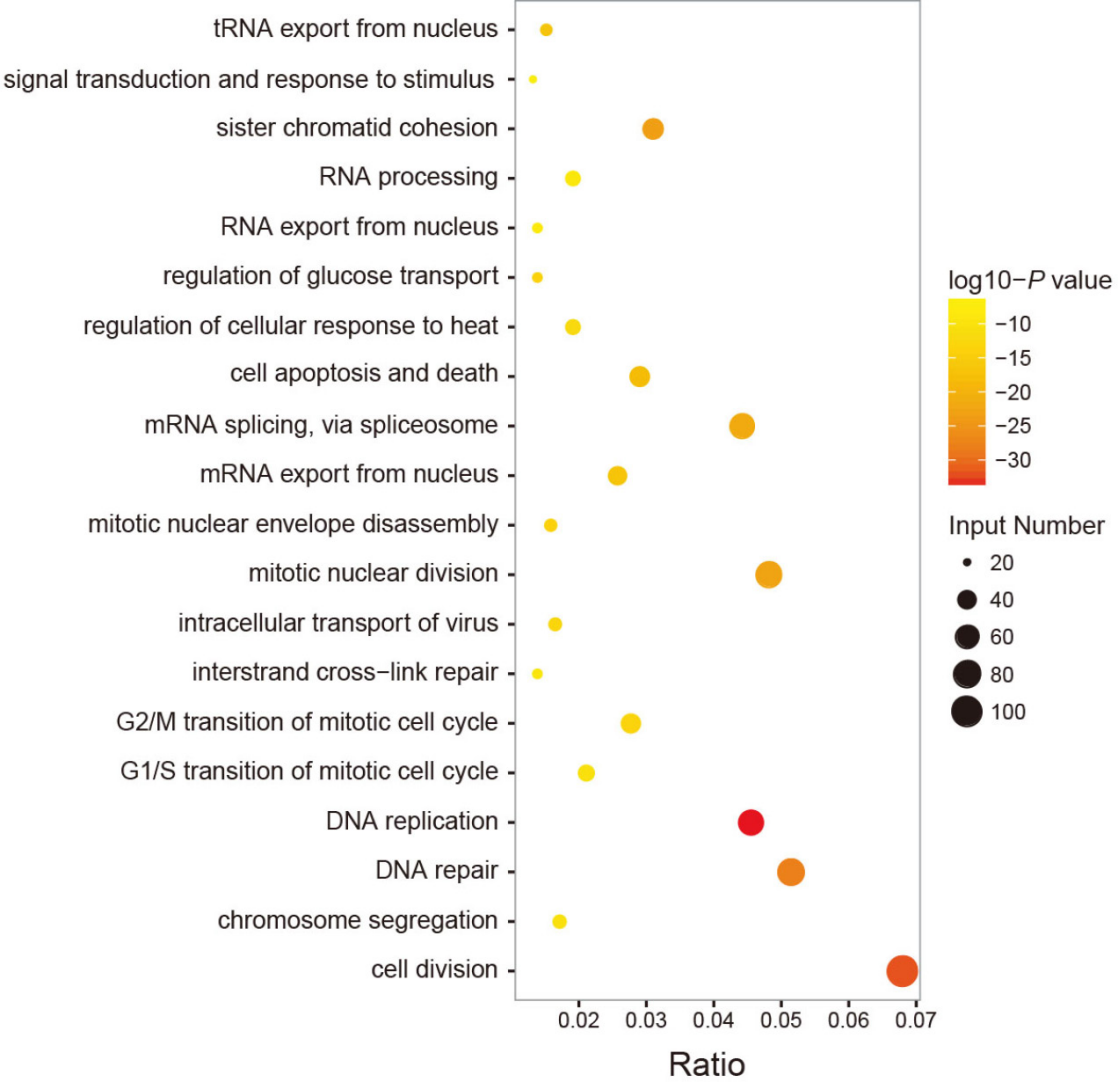
Functional enrichment analyses of the protein-coding genes co-expressed with the four prognostic lncRNAs (**A**) The functional enrichment map of GO terms. *Each node* represents a GO category. An *edge* represents the overlap of the shared genes between connecting terms. *Node size* represents the number of gene in the GO terms. *Color intensity* is proportional to enrichment significance.

We investigated the potential functional roles of the four prognostic lncRNAs in CRC. Spearman correlation coefficients were calculated between lncRNAs and protein-coding genes using the expression profiles of 344 CRC patients. A total of 732 protein-coding genes were positively correlated with either of the four lncRNAs (Spearman correlation coefficient > 0.6). Functional enrichment analyses indicated that these protein-coding genes were significantly enriched in 20 GO categories (*P-value* of < 0.01, Figure 5). These functionally enriched GO categories included assembly and disassembly of protein and macromolecules, transcription, signal transduction and response to stimulus, cell apoptosis and death, metabolic and catabolic process, cell cycle, DNA replication and DNA repair, etc. The result suggested that the four prognostic lncRNAs may participate in CRC tumorigenesis through regulating protein-coding genes to influence CRC-related biological pathways.

## DISCUSSION

Great efforts have been devoted to detect prognostic biomarkers for CRC at protein-coding and non-coding genes [21, 22, 26, 27]. Mounting evidence suggested that expression changes of lncRNAs are implicated in tumorigenesis by acting as tumor oncogenes or suppressor [8, 28]. Moreover, dysregulation of lncRNA has been measured in various cancer types, highlighting their potential roles as novel independent biomarkers for cancer prognosis [10, 29–32]. Some works have identified potential prognostic lncRNA signatures to predict overall survival in many cancer types, such as glioblastoma, lung cancer, etc. [15, 18]. However, the prognostic power of lncRNA signature for predicting survival in patients with CRC has still not been investigated.

Up to date, many lncRNAs have been discovered in human over the past decades [33]. However, only few of them are well characterized in human cancers. Among these four lncRNAs, *SPRY4-IT1* and *LINC01133* have been reported to be prognostic factors in patients with CRC [34, 35]. In this work, we identified that four lncRNAs are significantly associated with CRC patients’ survival and established a four-lncRNA signature for the prediction of survival. The result suggested a competitive performance of four-lncRNA signature for predicting survival. This finding can be validated by using TCGA testing set and another independent dataset, which demonstrated the reliability and reproducibility of the four-lncRNA signature for predicting CRC patients’ survival. Further stratified analyses after controlling for age and tumor stage showed that the prognostic power of the four-lncRNA signature was independent of other clinical variables for survival prediction of patients with CRC.

Previous studies documented that lncRNAs participated in biological processes by positively regulating protein-coding genes involved in the same processes. It is possible to predict lncRNA biological functions based on their co-expressed protein-coding genes [36–38]. Here, we performed GO enrichment analyses for lncRNA co-expressed protein-coding genes. The results demonstrated the important functional roles of the four prognostic lncRNAs in CRC tumorigenesis.

Taken together, we performed a comprehensive analysis for lncRNA expression profiles and corresponding clinical information in CRC patients. Our work identified that four prognostic lncRNAs were significantly associated with CRC patients’ survival. A four-lncRNA signature was established to effectively predict patients’ survival. The four-lncRNA signature might function as novel independent biomarkers for CRC prognosis. Our work gains insight into the understanding of the molecular mechanism of CRC.

## MATERIALS AND METHODS

### CRC datasets and clinical information

CRC lncRNA data and corresponding clinical information were downloaded from TCGA data portal. A total of 344 CRC patients were included in this work after removal of patients without clear clinical information. The lncRNAs derived from TCGA were annotated based on GENCODE database [39] to reduce redundant. The lncRNA expressions were defined as those with an average Fragments Per Kilobase of transcript per Million fragments mapped (FPKM) ≥ 0.1. The lncRNAs expression profiles were normalized by log2 transformed. At last, a total of 14,467 lncRNAs were enrolled in 344 CRC patients.

### Identification of prognostic lncRNA signature

We randomly divided CRC patients into a training set (*n* = 172) and a testing set (*n* = 172). In this training set, the association between the lncRNA expression and the overall survival of CRC patients was evaluated using a univariate Cox regression analysis. The lncRNAs that are significantly associated with the overall survival of CRC patients were identified based on the threshold of *P-value* < 0.01. Next, those selected lncRNAs were subjected to a multivariate Cox regression analysis. We established a risk score formula according to the lncRNA expression, weighted by the regression coefficients derived from the multivariable Cox regression analysis. Then, CRC patients in the training set can be divided into high-risk or low-risk groups by using the median risk score as a threshold.

The survival differences between high-risk and low-risk group in each dataset can be evaluated by the Kaplan-Meier analyses. Multivariate Cox regression and stratified analyses were carried out to evaluate whether the prognostic power of the four-lncRNA signature was independent of other clinical variables. The receiver operating characteristic (ROC) curve analyses were performed to evaluate the competitive performance for overall survival prediction. Area under the ROC curve (AUC) values were calculated. All analyses were performed using R package.

### Functional enrichment analyses

Since lncRNAs are always co-expressed with neighboring coding genes, we calculated spearman correlation coefficients to evaluate co-expression relationships between lncRNAs and protein-coding genes. Functional enrichment analyses for those co-expressed protein-coding genes were performed using the DAVID software [40, 41]. Gene Ontology (GO) categories with a *P-*value of < 0.01 were considered as significantly enriched function annotations.
